# The Course of Habituation of the Proboscis Extension Reflex Can Be Predicted by Sucrose Responsiveness in *Drosophila*

**DOI:** 10.1371/journal.pone.0039863

**Published:** 2012-06-26

**Authors:** Münire Özlem Çevik, Ahmet Erden

**Affiliations:** Abant Izzet Baysal Universitesi, Bolu, Turkey; Max-Planck-Institut für Neurobiologie, Germany

## Abstract

The proboscis extension reflex (PER) is triggered when insects’ gustatory receptors contact appetitive stimuli, so it provides a behavioral readout for perceptual encoding of tastants. Research on the experience dependent modulation of PER in *Drosophila* has been hindered by the difficulty of obtaining reliable measures of memory-driven change in PER probability in the background of larger changes induced by physiological state. In this study, we showed that the course of PER habituation can be predicted by the degree of sucrose responsiveness in *Drosophila*. We assessed early response parameters, including the number of proboscis extensions and labellar movements in the first five trials, the trial to start responding, and the trial to make the first stop to quantify responsiveness, which predicted the upcoming pattern of both the short-term and 1 hour memory of PER habituation for individual flies. The cAMP signaling pathway mutant *rutabaga* displayed deficits in attunement of perceptual salience of sucrose to physiological demands and stimulus-driven sensitization.

## Introduction

Habituation refers to the reduction in the probability or intensity of a response that occurs upon the repetition of the eliciting stimulus. Often considered as the simplest form of learning, it underlies the selection process that allocates attention to relevant stimuli, while diminishing responsiveness to the redundant background [1]–[2]. Insects respond to appetitive stimuli with an extension of their mouthparts called the proboscis, habituation of which has been reported several years ago for the housefly [3]. More recent studies showed that in honeybees, repeated stimulation of the antennae by sucrose not only causes a short-term habituation of the proboscis extension reflex (PER), but may also increase its threshold for hours [4].

Experiments on the learning dependent change of PER threshold have been sparse in Drosophila [5]–[8], despite the fact that this reflex may provide a tractable model for studying perception and plasticity. The probability of PER can be used as a behavioral readout for taste discrimination, learning dependent modulation of appetitive value, or decision making so long as it quantitatively tracks a relevant tastant property (e.g., concentration or type of tastant). The difficulty stems from the fact that, in addition to stimulus properties, PER elicitation is modulated by physiological factors including hunger [9]–[11], nutrition [12] and arousal [13]–[15]. Appetitive memory experiments are conducted on food deprived animals because feeding-related behaviors are not otherwise performed [16]. However, food deprivation triggers multiple mechanisms with different dynamics that modulate PER threshold [17], which may interact or even interfere with the stimulus-driven activation of PER. For example, a recent study showed that food deprivation leads to an increase in the gain of appetitive transmission and sensitizes the PER [10]. The modulation of PER threshold by the internal state of the organism may therefore introduce confounds to the experiments if the probability of PER is assessed to quantify the perceptual encoding of tastant properties and its learning dependent modification.

In this study, we tested the habituation of the proboscis extension reflex to a high (600 mM) concentration of sucrose following short (1–4 hours) periods of food deprivation to understand how hunger, short-term habituation, and 1-hour habituation memory interact to modulate PER probability in *Drosophila*. In a habituation session that consisted of 20 sucrose presentations, we used the response parameters of the first five trials (i.e., number of PERs and labellar movements, trials to the first PER and trials to the first stop) to assess sucrose responsiveness, which predicted the upcoming pattern of habituation in individual flies. Finally, to understand the role of cAMP signaling in hunger and memory dependent modulation of PER probability, we also tested PER habituation in *rutabaga* flies that are deficient for the Ca^2+^/calmodulin dependent adenylyl cyclase.

## Materials and Methods

### Flies

Canton-S and *rutabaga^2080^*/*rutabaga^2080^*; *ry/ry* (on a Canton-S background) flies were provided by Scott Waddell.

### Food

Fly food is very important because it changes the response threshold to appetitive stimuli. We use the Bloomington stock center formula. The recipe for 1.1 L of fly medium is: Cornmeal (Bünsa) 73.07 gr, Soy flour (Bünsa) 10 gr, Yeast (Dr. Oetker) 17.4 gr, Dry malt extract (British Diamalt) 46.16 gr, High fructose (55%) corn syrup (Cargill) 80 ml, Propionic acid (Sigma 99%) 4.82 ml, Agar (Roth) 5.5 gr.

### Fly Breeding and Maintenance

Fly stocks were kept in a Nüve ES 110 incubator under a 12 hrs light: 12 hrs dark cycle. The temperature and relative humidity (RH) in the incubator were set to 24°C and 70–75%, respectively. 3–5 females were allowed to lay eggs in 200 ml glass bottles containing 40 ml fly medium, and transferred to new bottles every other day because flies that grow in crowded bottles have lower PER thresholds. Male flies were collected within 2–3 hours of eclosion, maintained in groups of 10–15 until the experiment, and discarded if a female was placed in the bottle by mistake. The experiment was done when the flies were 4 days old. Flies were transferred to fresh food bottles the night before the experiment. An opaque paper cylinder was wrapped around the bottles to reduce visual stimulation, and extensive care was employed to prevent stimulation that could trigger excitation or activity before or during the experiment [14].

### Habituation Tests

The experiments were conducted in a temperature and humidity-controlled, red-illuminated 15 m^3^ room. The flies were removed from the incubator at 3–4 hours, and tested between 4–8 hours of their circadian time. Flies were briefly cold-anesthesized and inserted into 2–20 µl pipette tips with only the head and the prothoracic legs exposed [6]. They were moved immediately to a closed, non-illuminated, acclimatized (21–22°C, 90–95% RH) 60×40×30 cm glass chamber where they were kept under conditions of minimal stimulation (including air current) that could trigger activity, or excitation. The top lid of the chamber was wire mesh covered with cotton material to allow vaporization without dripping. Humidity was provided by Tefal ultrasonic cool moisture humidifiers.

Habituation was conducted using a Leica S6D stereomicroscope. Flies were discarded if they extended their probosces in response to water before the experiment. The habituation session consisted of 20 presentations of 600 mM sucrose to one of the prothoracic tarsi at 5 s inter-stimulus intervals. 5 ml insulin syringes were used for presenting sucrose. The tarsus was rinsed with water after each sucrose presentation. Flies were not allowed to ingest sucrose or water throughout the experiment, and they were discarded if their antennae, labellar receptors, or the non-habituated frontal tarsus touched sucrose at any time during the experiment. Flies were rehabituated in the same order that they were trained, and rehabituation started exactly 1 hr following the termination of training. Flies of the memory and control groups were collected from the same vials, and run in mixed order on every day of the experiment.

A computer program, written in Borland C++ was used to give an auditory signal at the programmed inter-stimulus-interval. Using a numeric code, the response was recorded as being no PER, PER without labellar movements, or PER with labellar movements, which correspond to 0–2, 3–5, and 6, respectively, on Dethier’s 6 point scale [18].

**Figure 1:**
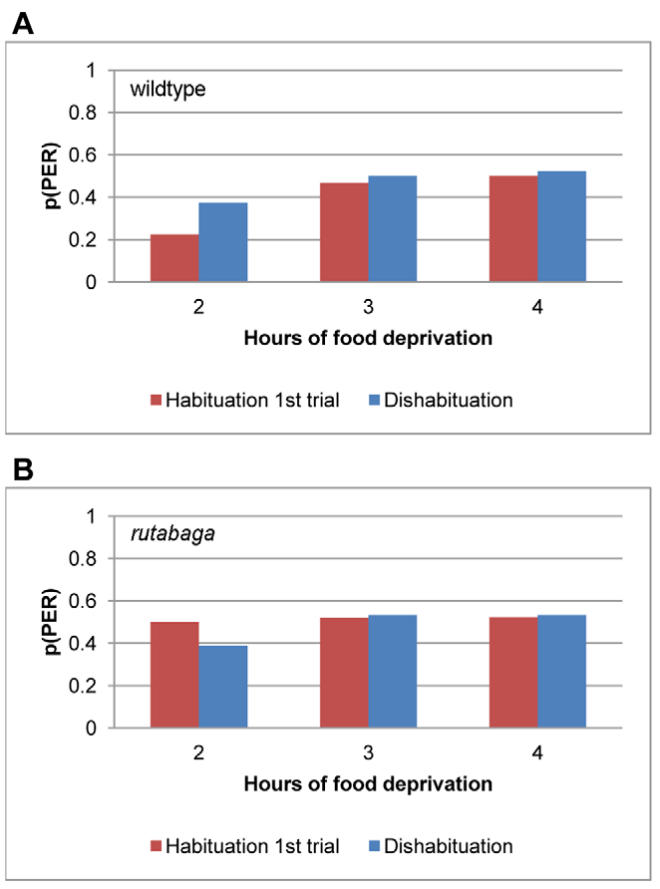
Dishabituation. The probability of proboscis extension to the first stimulus of the habituation session (red) or the dishabituating stimulus presented to the contralateral tarsus at the end of the session (blue) for the wildtype flies (A) and *rutabaga* (B) following 2–4 hours of food deprivation.

In order to test for the effects of fatigue, we presented a dishabituating stimulus to the contralateral foreleg at the end of the habituation session. Because the stimulation of the contralateral tarsus may interfere with memory formation, we did not apply it to the flies that were later to be rehabituated [4]. All of the flies that were trained at 1 hfd would be rehabituated later, so the dishabituation test was conducted at 2–4 hfd only. Figure 1 shows that the probability of dishabituation was not different from the probability of PER to the first stimulus during the habituation test for the same individuals for either the wildtype (χ^2^ (1) = 1.06, p<.30), or *rutabaga* flies (χ^2^(1) = .04, p<.85). Further, the probability of dishabituation was not different between the two genotypes (χ^2^(1) = .25, p<.61), indicating that the observed pattern of change in PER probability is not likely to have resulted from effector fatigue for either genotype.

### Statistical Analyses

Data were analyzed using SPSS 17. The effect of the period of food deprivation was analyzed with a one-way ANOVA with the total number of PERs emitted in the session (totalPER) as the dependent variable. For each fly, we calculated 4 early response indices based on the first 5 trials of the session: Number of PERs (fivePER), number of labellar movements (fiveLAB), the trial on which the first PER appeared (firstPER), and the trial after which a PER was skipped after emitting at least one PER (firstSTOP). In order to understand the effects of early sucrose responsiveness, the *de novo* habituation data were collapsed across 1–4 hours of food deprivation, and analyzed using a two-way ANOVA with the early response index and the period of food deprivation as between-subjects factors, and totalPER as the dependent variable. Because firstPER, firstSTOP and fiveLAB could be defined for the responsive flies only, non-responsive flies were excluded from their analyses. A Scheffe analysis was performed after the ANOVA tests. The memory effect was analyzed using a one-way ANOVA. The relative frequency distributions were analyzed using the Pearson chi-square test. Two sided probability values from a Fisher’s exact test were used for trial-by-trial comparisons of the habituation curves. Other details are indicated in the text as necessary.

## Results

### *rutabaga* is Not Deficient in Short-term PER Habituation, but Shows a Defect in Hunger-driven Attunement of PER Threshold

**Figure 2:**
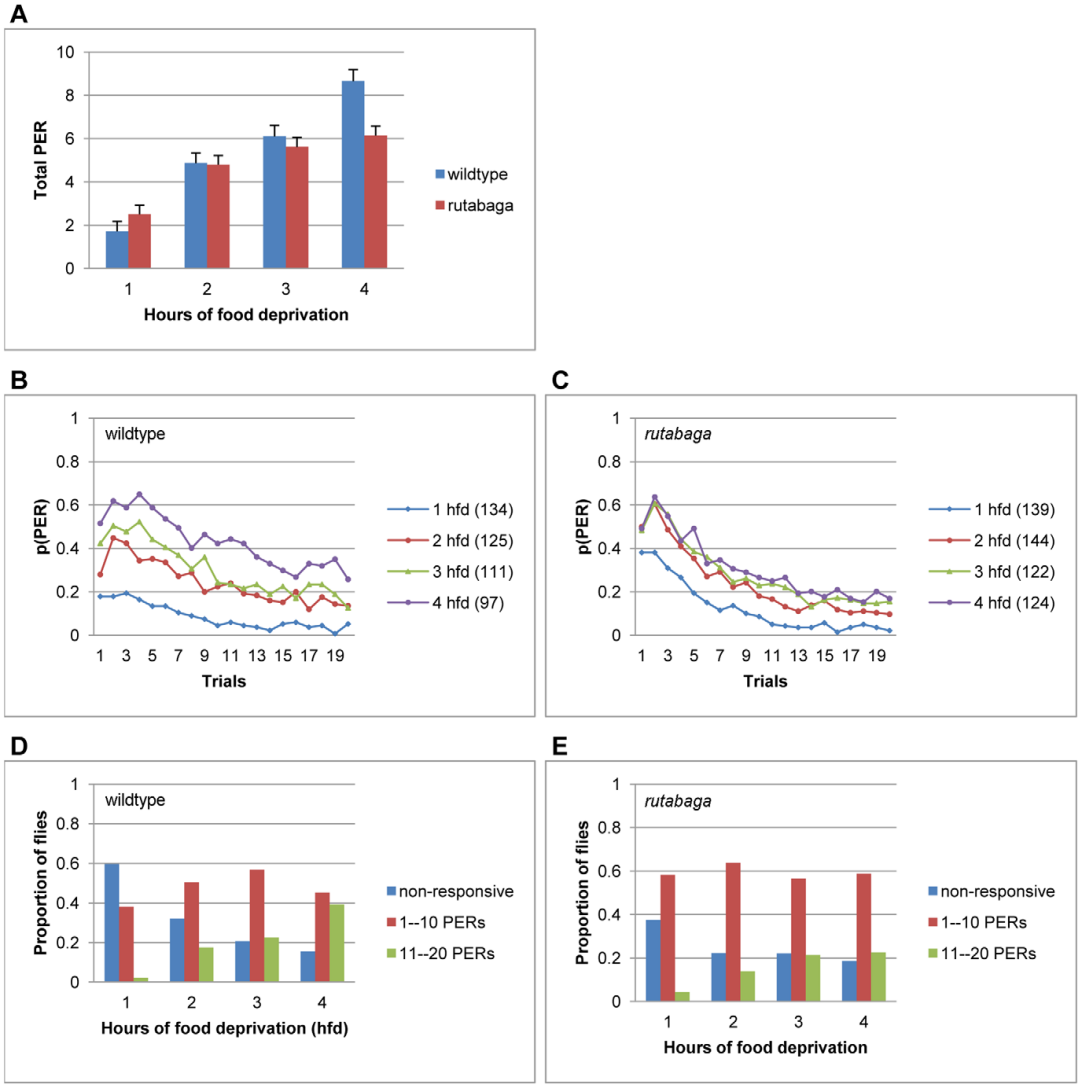
The effects of the period of food deprivation on PER habituation. **A.** Total number of PERs produced during the habituation session at 1–4 hours of food deprivation. **B-C.** PER habituation for the wildtype flies (B) and *rutabaga* (C) following 1–4 hours of food deprivation. Numbers in parentheses indicate sample size. **D-E.** The relative frequency distribution of totalPER scores at 1–4 hours of food deprivation for the wildtype flies (D) and *rutabaga* (E).

In order to understand the effects of hunger on habituation, we tested the flies following 1, 2, 3, or 4 hours of food deprivation (hfd). For the wildtype flies, the total number of proboscis extensions emitted during the habituation session (totalPER) increased (F(3, 463) = 34.87, p<.001, η^2^ = .18, Figure 2a, blue bars), and the habituation curves shifted upward (Figure 2b) with the period of food deprivation. The hunger-driven change in totalPER was significant between 1 to 2 hfd (p<.001) and 3 to 4 hfd (p<.007), but not 2 to 3 hfd (p<.35). In an attempt to understand how response profiles of individual flies changed across 1–4 hfd, we plotted the proportion of flies that were non-responsive, or responded to make either 1–10 or 11–20 totalPERs (Figure 2d). At 1 hfd, 60% of the wildtype flies were non-responsive throughout the session, which decreased to 32% by 2 hfd (χ^2^ (1) = 19.96, p<.001, blue bars). In contrast, the proportion of flies that produced more than 10 PERs increased between 1 and 2 hfd (χ^2^ (1) = 17.50, p<.001) and 3 and 4 hfd (χ^2^ (1) = 6.8, p<.009, green bars).

Next, we analyzed the effects of hunger on PER habituation in *rutabaga* flies that are deficient for the Ca^2+^/calmodulin activated adenylate cyclase. For *rutabaga,* although totalPER increased significantly with the period of food deprivation (F(3, 525) = 14.08, p<.001, η^2^ = .07, Figure 2a), a Scheffe analysis confirmed that it did not change after 2 hfd (p<.185). The wildtype flies and *rutabaga* produced similar numbers of totalPERs on average (F (1, 988) = 3.13, p<.08, η^2^ = .00), indicating that *rutabaga* did not show a deficiency in short-term PER habituation *per se* (Figure 2c). However, the genotype X deprivation period interaction reached significance (F (3, 988) = 4.49, p<.004, η^2^ = .01), because the totalPER scores of *rutabaga* started higher at 1 hfd but failed to increase between 3–4 hfd. Therefore, for *rutabaga*, not only the enhancement of PER by hunger, but also its suppression during satiation failed to occur to the same extent that they had for the wildtype flies. For example, at 1 hfd 63% of *rutabaga* emitted at least 1 PER, whereas roughly the same majority of wildtype flies had remained completely non-responsive (compare Figures 2d and e). A chi-square analysis confirmed that, at 1 hfd, *rutabaga* had a lower probability of remaining non-responsive (χ^2^(1) = 13.56, p<.001, blue bars), and a higher probability of responding at least once (χ^2^(1) = 11.16, p<.001, red bars) relative to the wildtype flies. Notice that the failure to suppress responding during satiation was independent of memory, because it was evident on the first trial of the session: Relative to the wildtype flies, *rutabaga* exhibited a higher probability of PER on the first trial at 1 (χ^2^(1) = 13.77, p<.001, compare Figures 2b and c, blue curves) and 2 hfd (χ^2^(1) = 13.52, p<.001, red curves) when the flies were relatively sated, but not at 3 (green curves) or 4 hfd (purple curves).

### Response Characteristics Early in the Session Predict Variability in Habituation

Next, we wanted to know whether early response parameters can be used to predict the variability in habituation.

**Figure 3:**
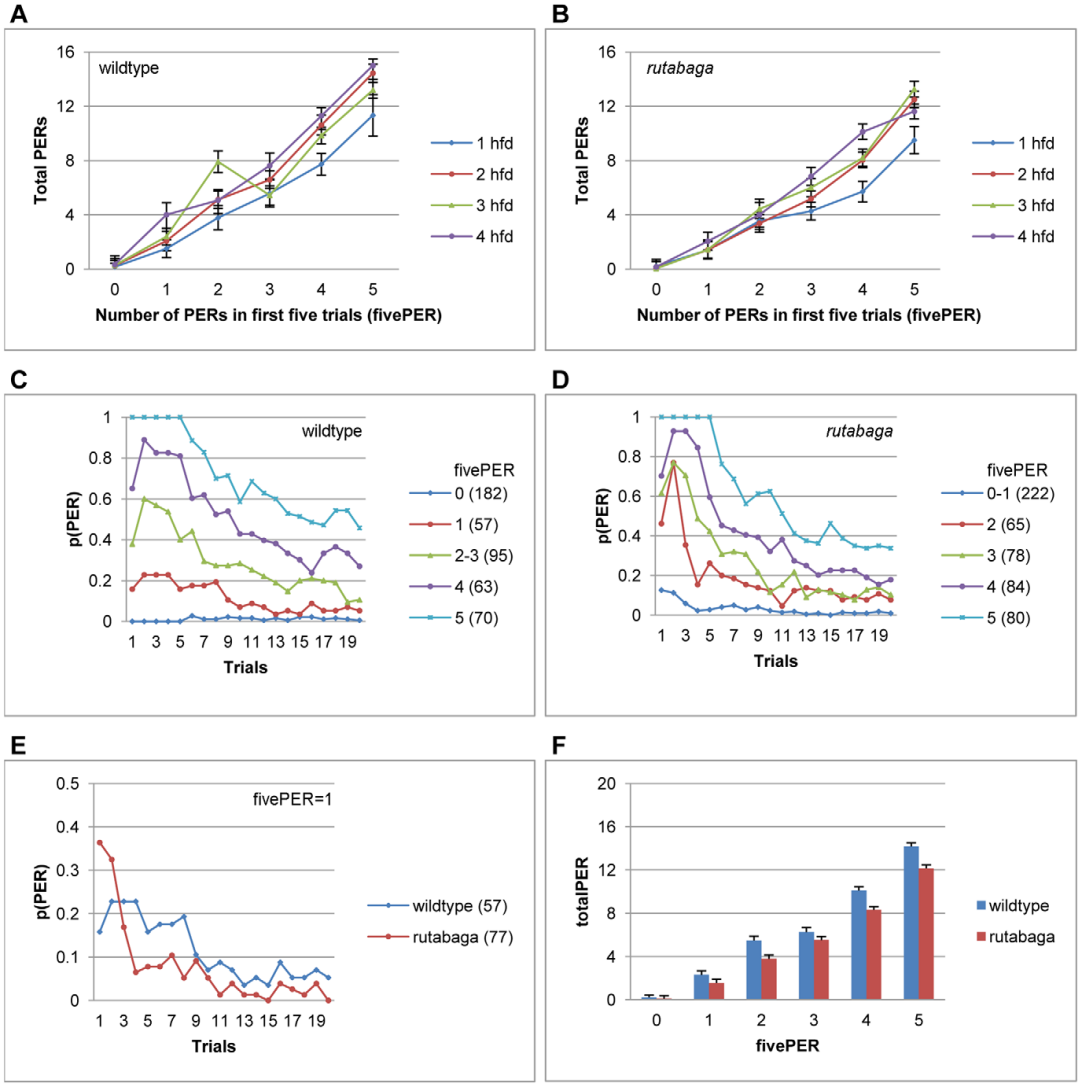
The number of PERs produced during the first five trials predicts the upcoming pattern of habituation. **A-B.** TotalPER increases in proportion with fivePER for the wildtype flies (A) and *rutabaga* (B) between 1–4 hfd. **C-D.** Habituation curves for homogenous subsets of fivePER (Scheffe) for the wildtype flies (C) and *rutabaga* (D). **E.** PER habituation for the wildtype flies and *rutabaga* that emitted 1 PER during the first five trials (fivePER = 1). **F.** Summary graph showing totalPER for different fivePER scores. Data collapsed across 1–4 hfd in C-F. Numbers in parantheses indicate sample size.

We first analyzed whether the number of PERs produced within the first five trials (fivePER) was predictive of the total number of PERs produced in the whole session (totalPER). Figure 3f shows that totalPER increased in proportion to fivePER for the wildtype flies, yielding a highly significant effect (F(5, 443) = 201.54, p<.001, η^2^ = .70). The ratio between fivePER and totalPER, and hence the slope of the function relating the two variables increased slightly with each additional deprivation hour (Figure 3a), indicating that as the period of food deprivation increased, flies that started out with similar responsiveness exhibited progressively higher response tendencies during the rest of the session (Figure 3c). When we fitted a linear equation, the slope and the y-intercept of the functions relating totalPER to fivePER varied between 2.2–2.8, and −.49−.47, respectively. That is, the average totalPER of the wildtype flies was 2 to 3 times as large as their fivePER values between 1 to 4 hfd. Under a two-way ANOVA, the proportion of variance in totalPER accounted for by fivePER (.70) was 15 times as large as that explained by the period of food deprivation (F(3,443) = 6.92, p<.001, η^2^ = .05), or the food deprivation X fivePER interaction (F(15, 443) = 2.01, p<.014, η^2^ = .06). Therefore, on average, the totalPER of a fly was more similar to those of flies that showed equal responsiveness during the first five trials irrespective of the period of food deprivation, than to those of flies that have been food deprived for the same amount of time. The proportion of flies that made 0 fivePER decreased (χ^2^ (3) = 66, p<.001), and those that made 4 (χ^2^ (3) = 9.7, p<.021) and 5 (χ^2^ (3) = 38.3, p<.001) fivePER increased significantly with the period of food deprivation. Hence, the hunger-driven increment in totalPER can be explained mainly in terms of the increase in the relative frequency of flies that show high initial responsiveness, and to a lesser extent by the increment in the overall tendency to respond given equal initial responsiveness.

### *rutabaga* Habituates Faster than the Wildtype Flies

Similarly for *rutabaga*, totalPER increased with fivePER, yielding a highly significant effect (F(5, 505) = 188.65, p<.001, η^2^ = .65, Figure 3f, red bars) that accounted for a proportion of variance 16 times as large as that explained by the period of food deprivation (F(3, 505) = 7.17, p<.001, η^2^ = .04) under a two-way ANOVA. The fivePER X deprivation hours interaction was also significant (F(15, 505) = 2.07, p<.01, η^2^ = .06), because the hunger effect was smaller for flies that exhibited lower fivePER scores. When we fitted a linear equation, the slope and the y-intercept of the functions relating totalPER to fivePER varied between 1.7 to 2.5, and −.9 to −.19, respectively (Figure 3b). That is, the average totalPER of the *rutabaga* flies was 2 to 2.5 times as large as their fivePER values between 1 to 4 hfd.

When equated for initial sucrose responsiveness, *rutabaga* showed a faster rate of habituation relative to the wildtype flies. Figure 3d shows the habituation curves for the homogenous subsets (Scheffe) of fivePER for *rutabaga.* For each value of fivePER, *rutabaga* displayed an equal or higher probability of PER relative to the wildtype flies during the first two trials, followed by a higher rate of habituation and a lower asymptote throughout the rest of the session. For a concrete example, we plotted the habituation curves of both *rutabaga* (red) and the wildtype flies (blue) that were equated for initial responsiveness at fivePER = 1 (Figure 3e). Given that each fly on Figure 3e made only one response during the first five trials, the relatively flat habituation curve of the wildtype flies across this period indicates that they showed an equal probability of starting to respond on trials 1–5. Further, because the probability of PER did not decrease significantly relative to its maximum (trial 2) until trial 10 (p<.03), some of the wildtype flies must have resumed responding after the first stop. *rutabaga,* on the other hand, showed a significantly higher probability of responding on the first trial (p<.01), followed by a faster rate of habituation: The drop in *rutabaga*’s PER probability was significantly lower than its own maximum by trial 3 (p<.01), and lower than that of wildtype flies by trial 4 (p<.009). These results indicate that *rutabaga* both started and stopped responding sooner and had a lower incidence of resuming responding after the first stop.

### Trials to First PER

The trial to make the first PER (firstPER) can be taken as an index of the perceptual salience of sucrose. The probability of responding on the first trial, in particular, is a purely perceptual measure that is not confounded by the effects of stimulus repetition.

**Figure 4:**
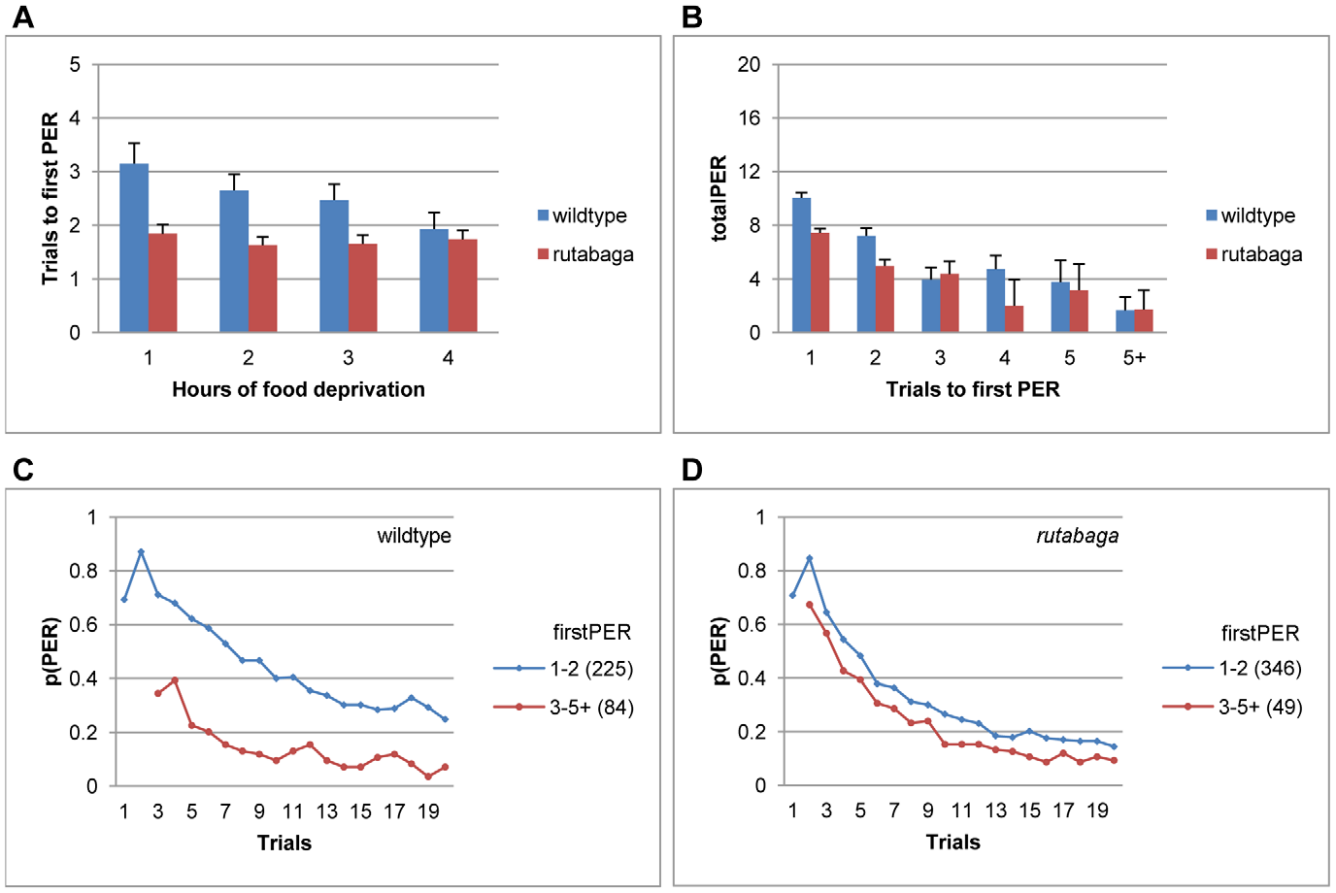
Trials to first PER predict the upcoming pattern of habituation. **A.** Average firstPER following 1–4 hfd. **B.** Average totalPER scores for the wildtype flies and *rutabaga* that started responding on trials 1–5+. **C-D.** Habituation curves for homogenous subsets of firstPER (Scheffe). Habituation curves of *rutabaga* are plotted in accordance with the subsets identified for the wildtype flies for comparability. Data are collapsed across 1–4 hfd in B-D. Numbers in parantheses indicate sample size.

firstPER could be defined for the flies that emitted at least 1 PER during the habituation session, so the non-responsive flies were excluded from the analyses. Although the wildtype flies began responding earlier as the period of food deprivation increased, the reduction in average firstPER was not significant (F(3, 305) = 2.23, p<.08, η^2^ = .02, Figure 4a, blue bars), because 65–80% of all responsive flies started responding within the first two trials between 1–4 hfd, respectively. The variability in firstPER was predictive of totalPER irrespective of the period of food deprivation. Wildtype flies that started to respond later completed the session with lower totalPER values (F(5, 285) = 16.7, p<.001, η^2^ = .23, Figure 4b, blue bars), and displayed a faster rate of habituation following their first PER (Figure 4c). For example, average totalPER was 10.05±.39 and 3.97±.9 for the flies that started responding on the first, and the third trial, respectively, so the difference between the totalPERs of the two groups was roughly three times as large as the difference between their firstPERs. Figure 4c shows the habituation curves for the homogenous subsets of firstPER identified by a Scheffe analysis. Although an early response onset (i.e., high sucrose salience, Figure 4c, blue curve) by itself was not a reliable predictor of a failure to habituate, a late response onset (i.e., low sucrose salience) was a reliable predictor of rapid habituation.

For *rutabaga*, the trial to start responding did not change at all with the period of food deprivation (F(3, 391) = .34, p<.8, η^2^ = .00, Figure 4a, red bars), and a noticeable 85–92% of all rutabaga started responding within the first two trials across 1–4 hfd, respectively. The absence of late onset responding in *rutabaga* resulted in a small but significant decrement in firstPER relative to that of the wildtype flies (F(1, 702) = 21.99, p<.001, η^2^ = .03).

Although *rutabaga* that started responding earlier completed the session with higher totalPER scores (F(5, 372) = 7.64, p<.001, η^2^ = .09), irrespective of the period of food deprivation (F(14, 372) = .55, p<.9, η^2^ = .02, Figure 4b, red bars), firstPER accounted for a lower proportion of variance in totalPER (.09) in *rutabaga* relative to the wildtype flies (.23). Correspondingly, when plotted for different values of firstPER, the habituation curves diverged to a lesser extent for *rutabaga* than they had for the wildtype flies (compare Figures 4c and d). The Scheffe analysis identified only one homogenous subset of firstPER for *rutabaga*, and Figure 4d was plotted in accordance with the subsets of the wildtype flies (Figure 4c) for comparability.

### Trials to First STOP

Next, we asked whether the number of trials to make the first stop (firstSTOP) is predictive of the pattern of habituation. Because flies start responding on different trials, making a stop on a given trial provides rather imprecise information about the overall responsiveness of a fly. The analysis of firstPER showed that flies that started responding after the 2^nd^ trial displayed limited variability with respect to overall responsiveness. Therefore, for a precise understanding of the predictive value of the latency to first stop on the course of habituation, we analyzed the data from flies that started responding on the first two trials only, which comprised 73 and 88% of all responsive wildtype and *rutabaga* flies, respectively.

**Figure 5:**
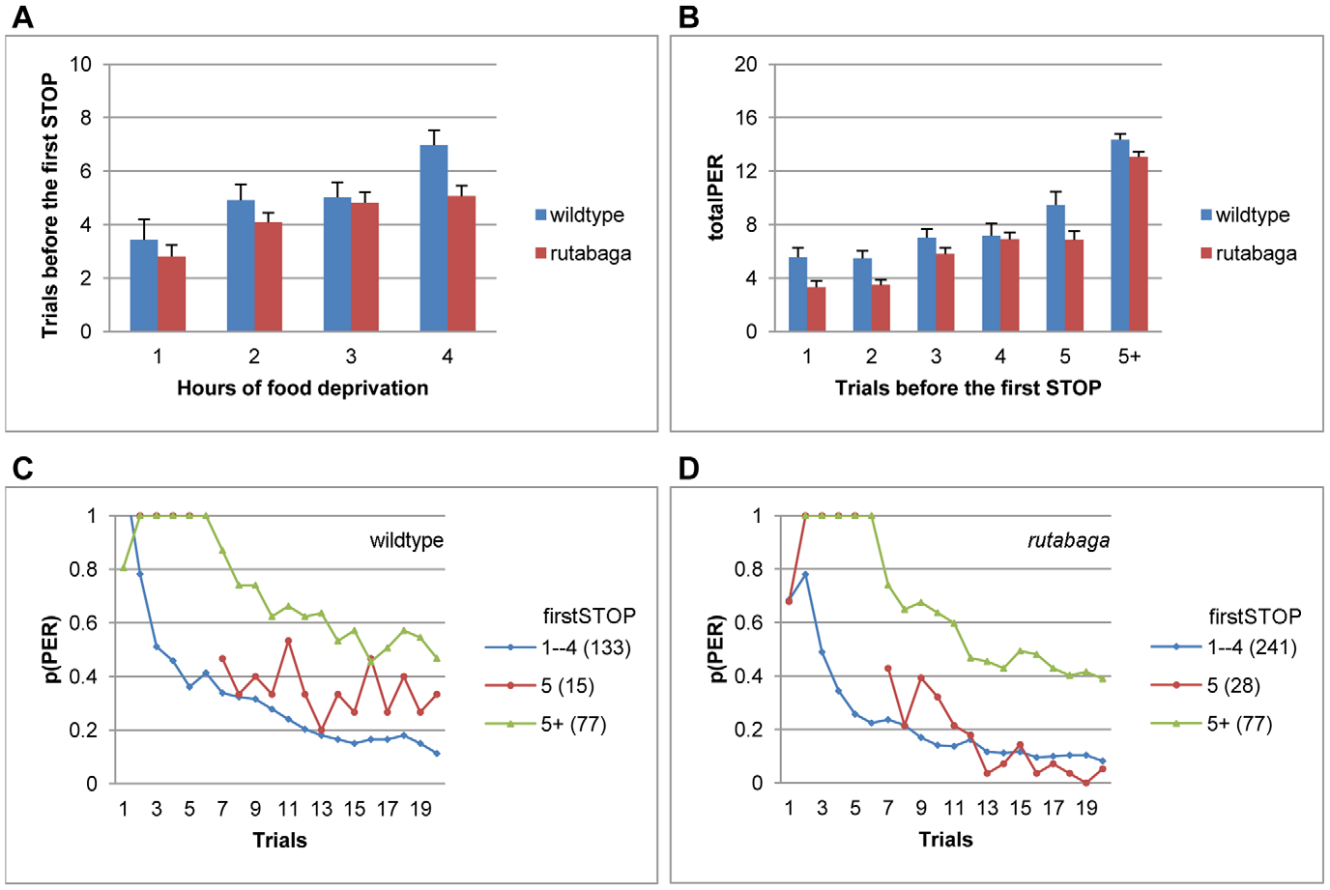
The trial after which the flies make their first stop predicts the upcoming pattern of habituation. **A.** Average firstSTOP for the flies that started responding on the first two trials following 1–4 hfd. **B.** Average totalPER increases with firstSTOP. **C-D.** Habituation curves for homogenous subsets of firstSTOP (Scheffe). Habituation curves of *rutabaga* are plotted in accordance with the subsets identified for the wildtype flies for comparability. Data are collapsed across 1–4 hfd in B-D. Numbers in parantheses indicate sample size.

In general, the first uninterrupted sequence of PERs that occurred before the first stop was also the longest, so the pattern of change in firstSTOP with the period of food deprivation (Figure 5a) closely resembled that of totalPER (Figure 2a; totalPER scores were lower because the non-responsive flies were not included in the analysis of firstSTOP). The variability in firstSTOP was highly predictive of totalPER for the wildtype flies (F(5, 201) = 29.85, p<001, η^2^ = .43), irrespective of the period of food deprivation (F(15, 201) = .6, p<.86, η^2^ = .03, Figure 5b). For example, if a wildtype fly made an early stop before the fifth repetition of the sucrose (Figure 5c, blue curve), it proceeded to display robust habituation, emitting 6.14±.35 PERs on average. In contrast, if flies did not stop until after the sixth trial, they continued to respond with occasional remissions and produced 14.35±.41 PERs (green curve), which was significantly higher than the totalPERs of flies that stopped earlier (p<.001). Therefore, although an early response onset by itself is an ambiguous behavioral parameter with respect to the state of responsiveness, the latency of the first stop following an early onset can be used to predict the course of habituation.

The variability in firstSTOP was highly predictive of totalPER for *rutabaga* as well (F(5, 322) = 55.34, p<001, η^2^ = .46), irrespective of the period of food deprivation (F(15, 322) = 1.2, p<.27, η^2^ = .05, Figure 5b). Like the case with the wildtype flies, the failure to make a stop until after the sixth trial predicted a visibly slower rate of habituation to a higher asymptote in *rutabaga* (Figure 5d, green curve).

### Labellar Movements in the First Five Trials Predict Variability in Habituation

**Figure 6:**
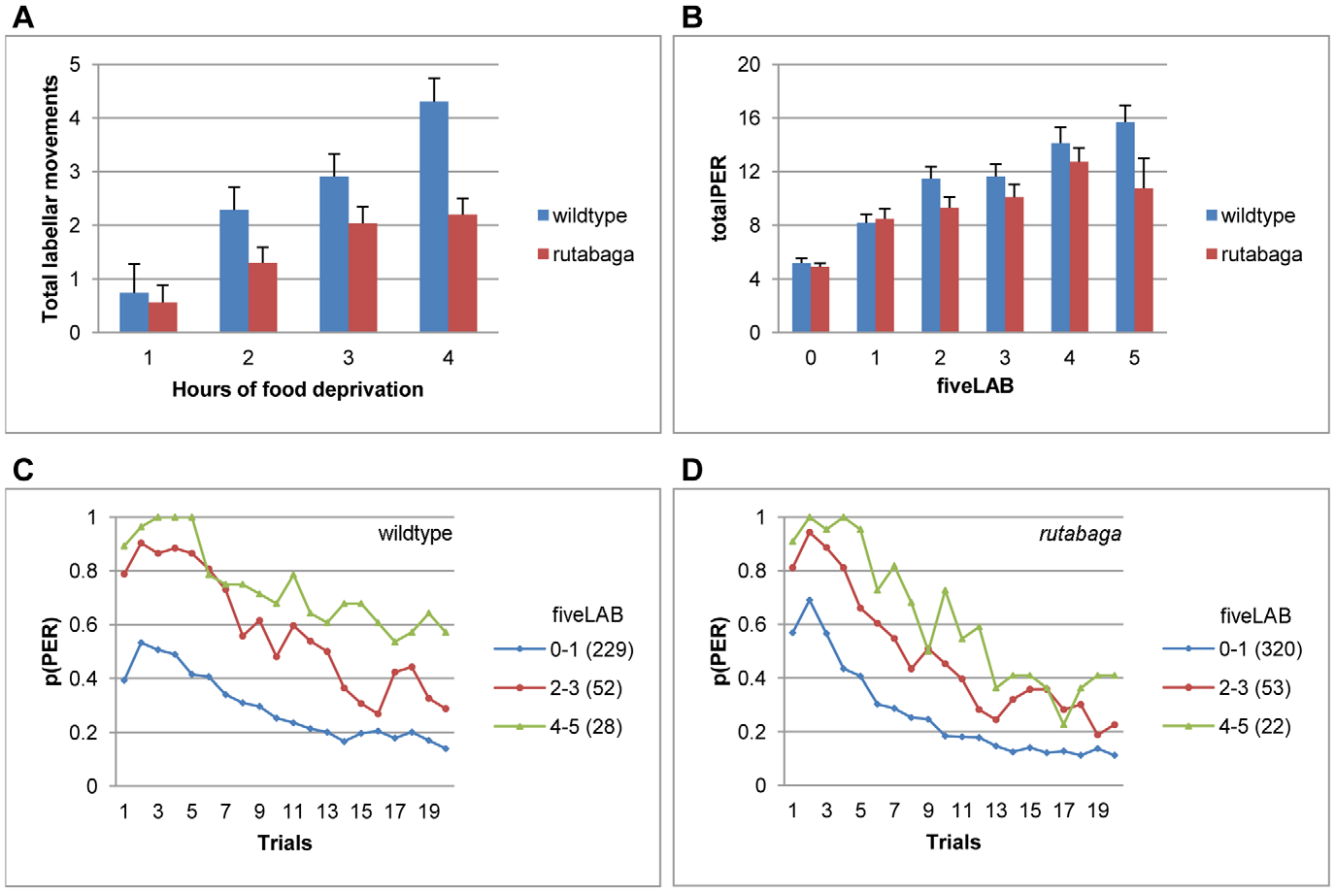
Number of labellar movements emitted during the first five trials (fiveLAB) predicts upcoming PER habituation. **A.** Total number of labellar movements produced by the flies that emitted at least 1 PER between 1–4 hfd. **B.** TotalPER for the wildtype and *rutabaga* flies that produced 0–5 fiveLAB. **C-D.** Habituation curves for homogenous subsets of fiveLAB (Scheffe). Habituation curves of *rutabaga* are plotted in accordance with the subsets identified for the wildtype flies for comparability. Data are collapsed across 1–4 hfd in B-D. Numbers in parantheses indicate sample size.

Next, we wanted to understand if the topology of proboscis extension in the early trials is predictive of the pattern of habituation. Because labellar movements are observed only if the proboscis is already extended, non-responsive flies were excluded from the analysis. For the wildtype flies, the number of proboscis extensions that were accompanied by labellar movements increased with the period of food deprivation (F(3, 305) = 9.25, p<.001, η^2^ = .08, Figure 6a, blue bars). The majority of the wildtype flies failed to emit any labellar movements when they extended their probosces up to 3 hfd, and the proportion of responsive flies that did not show any labellar movements within the first five trials (fiveLAB) decreased from 80 to 43% between 1 to 4 hfd (χ^2^ (3) = 18.81, p<.001). Wildtype flies that emitted higher numbers of labellar movements during the first five trials (fiveLAB) completed the session with higher totalPER values (F(5, 287) = 16.77, p<.001, η^2^ = .23, Figure 6b, blue bars) irrespective of the period of food deprivation (F(13, 287) = .65, p<.83, η^2^ = .05). For the wildtype flies, the high incidence of labellar movements was the strongest predictor of a failure to habituate among all early behavioral indices. For example, 60% of all flies that emitted 4–5 labellar movements in the first five trials were still responding on the last trial of the session (Figure 6c, green curve). These results suggest that labellar movements have a higher threshold of modulation by hunger than PER, and their sensitization is a reliable predictor of high sucrose responsiveness.

For the *rutabaga* flies, although the total number of labellar movements emitted in the session increased with the period of food deprivation (3, 391) = 5.63, p<.001, η^2^ = .04), overall, the incidence of labellar movements was lower relative to the wildtype flies (F (1, 696) = 14.98, p<.001, η^2^ = .02, Figure 6a, red bars). The proportion of responsive flies that did not emit any labellar movements when they extended their probosces decreased from 86 to 61% between 1 to 4 hfd. At 4 hfd, 57% of the wildtype flies moved their labella at least once during the first five trials, whereas the same proportion of *rutabaga* failed to make any labellar movements (χ^2^ (1) = 6.36, p<.012). Further, very few *rutabaga* flies emitted 5 fiveLABs which is consistent with the attenuation of hunger-modulated sensitization of feeding reflexes in this mutant.

Higher numbers of labellar movements during the first five trials (fiveLAB) were predictive of higher totalPER scores (F(5, 372) = 14.02, p<.001, η^2^ = .16) irrespective of the period of food deprivation (F(14, 372) = .38, p<.98, η^2^ = .01, Figure 6b, red bars) for *rutabaga* as well. The homogenous subsets analysis showed that, for *rutabaga,* although the absence of labellar movements within the first five trials predicted higher rates of habituation, the exact number of labellar movements failed to make a difference when they were present. In Figure 6d, we plotted the habituation curves of *rutabaga* in accordance with the homogenous subsets of fiveLAB of the wildtype flies (Figure 6c) for comparability.

### The Effects of Hunger and 1-hour Memory on PER Threshold are not Additive

Savings in learning refers to the reduction in the number of trials required for behavioral change due to the enduring effects of previously formed memories. Under the current paradigm, if memory persists after the termination of the initial habituation session (training), savings would be expected to produce a faster decrement in PER probability upon repetition of the habituation test (rehabituation). The hunger-driven increment in PER probability over the period allocated to memory formation could complicate the assessment of savings, measurement of which can be reliable only in the background of equal sucrose responsiveness. Therefore, in order to assay savings in PER habituation, we compared the memory group’s rehabituation performance with that of control flies that were habituated *de novo* at the same hour of food deprivation.

**Figure 7:**
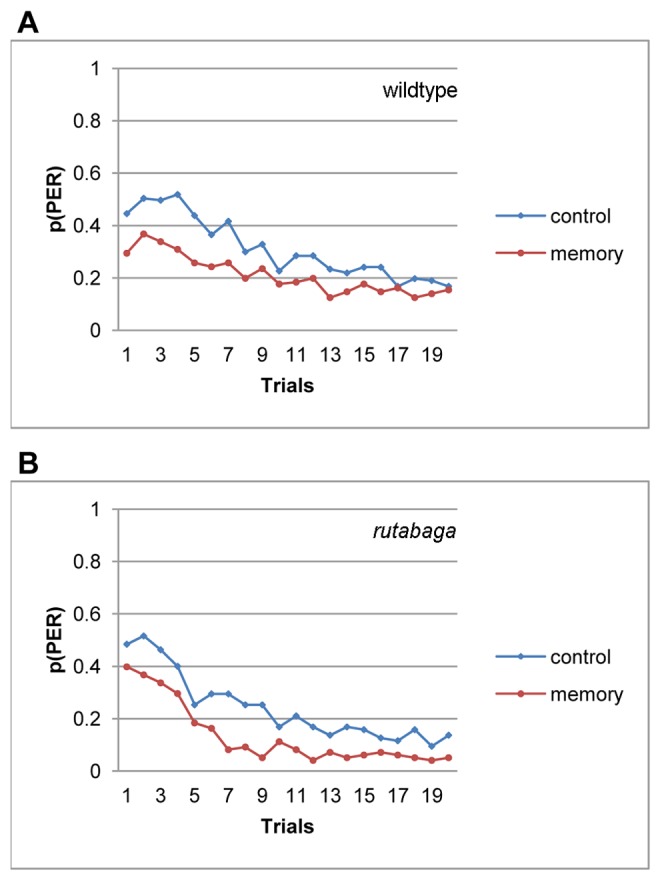
One hour memory of PER habituation. One hour memory of PER habituation for the wildtype flies (A) and *rutabaga* (B).

If the effects of memory and hunger on PER threshold were additive, a constant difference would be expected between the memory and control groups at different hours of food deprivation. However, the extent of savings changed with the period of food deprivation, suggesting a non-additive relation between hunger and memory in the modulation of PER threshold. For example, when we assayed 1-hour memory at 4 hfd, average totalPER scores of the memory and the control groups were not different (F(1,63) = .31, p<.51, η^2^ = .01), and the habituation curves overlapped extensively, suggesting that the memory effect, if existed at all, was overridden by hunger at this time (data not presented). Although the memory effect failed to reach significance at 2 (F(1, 96) = 1.93, p<.17, η^2^ = .02) or 3 hfd (F(1, 72) = 1.9, p<.17, η^2^ = .03) as well, a difference in the habituation curves of memory and the control groups was evident over the first 10 trials (data not presented). In particular, at 3 hfd, the difference between the totalPER scores of the memory and control groups yielded a marginal significance for the first 10 trials (F (1, 72) = 4.07, p<.047, η^2^ = .05). This was a small effect, explaining only 5% of the variance in totalPER. We ran 4 additional replications of the 1-hour PER memory test at 3 hfd, of which only one yielded a significant difference in totalPER of the memory and the control groups (F (1, 46) = 10.19, p<.003, η^2^ = .18), and three did not reach significance (p<.12). We merged the data from all 5 replications to achieve a large sample size for analyzing the dependence of 1 hour memory of PER habituation on sucrose responsiveness. The habituation curve of the memory group in the merged data set was shifted downward relative to that of the control group, revealing a small effect of 1 hour memory (F(1, 271) = 9.02, p<.003, η^2^ = .03, Figure 7a).

*rutabaga* flies have consistently been reported to be defective in short- and long-term memory under both associative (for a review see Davis, 2005) and non-associative (for a review see Engel and Wu, 2009) learning procedures. We found that although *rutabaga* failed to display savings due to 1-hour memory at 3 (F(1, 93) = 2.62, p<.11, η^2^ = .03), or 4 hfd (F(1, 61) = .03, p<.86, η^2^ = .00,), the memory effect was significant at 2 hfd (F(1, 81) = 4.13, p<.045, η^2^ = .05, data not presented). Like the case with the wildtype flies, the memory effect was small, and it accounted for only 5% of the variance in totalPER scores. An independent replication (F (1, 108) = 6.49, p<.012, η^2^ = .06) yielded similar results. When we merged the data from the two replications to attain a large sample to analyze dependence of 1-hour memory on sucrose responsiveness, the memory effect was significant (F(1, 191) = 10.72, p<.001, η^2^ = .05), and the rehabituation curve of the memory group was downshifted relative to control (Figure 7b).

### Wildtype Flies that Exhibit Low Sucrose Responsiveness Express 1 Hour Memory of PER Habituation

**Figure 8:**
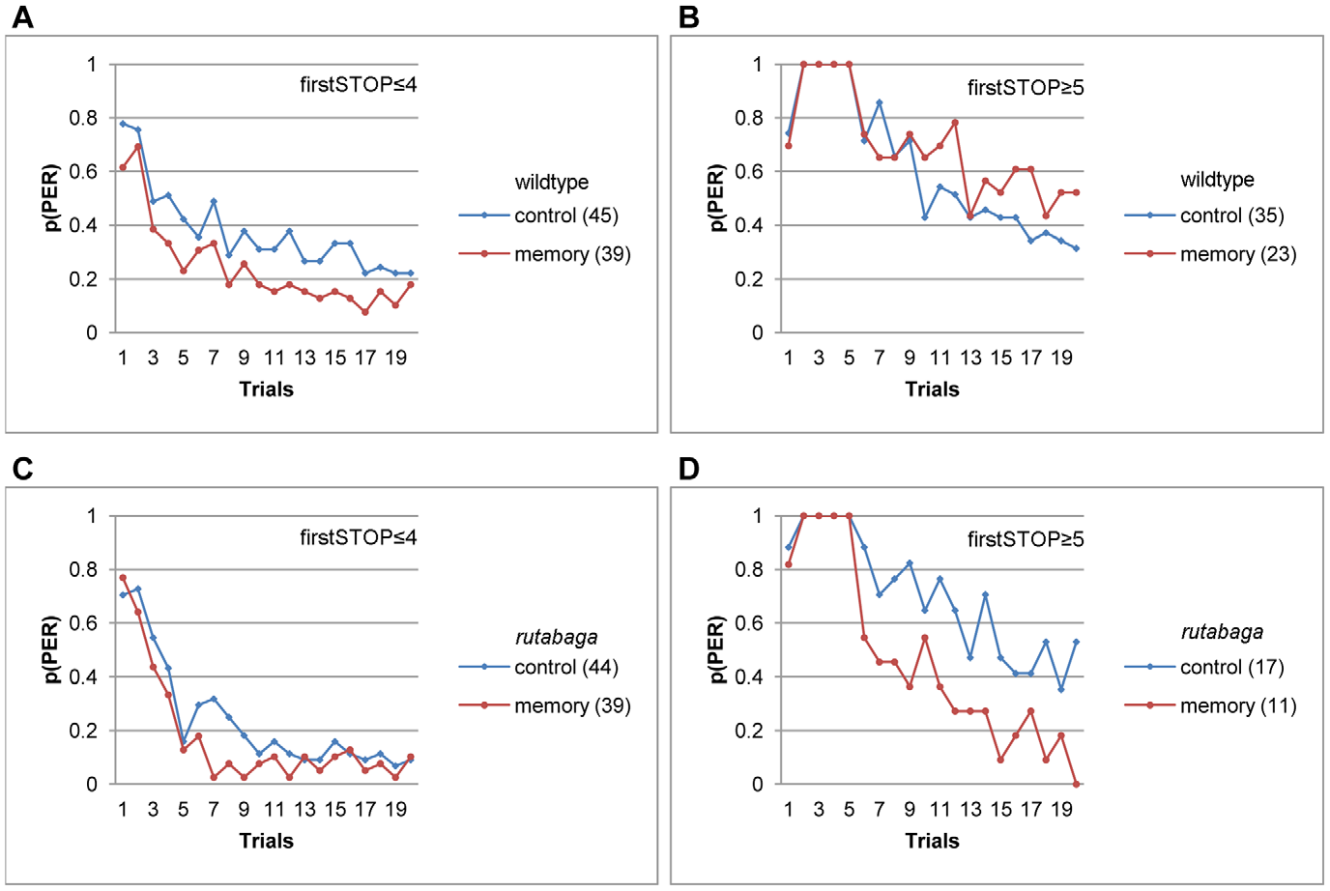
The trial to make the first stop is predictive of the expression of 1-hr memory of PER habituation . **A-B.** One hour memory of PER habituation for the wildtype flies that made their first stop before (A) or after (B) the fifth trial. **C-D.** One hour memory of PER habituation for *rutabaga* that made their first stop before (C) or after (D) the fifth trial.

When we compared the memory and control flies that were equated for their responsiveness using firstSTOP and fiveLAB, we found that 1 hour memory of PER habituation is expressed selectively by the wildtype flies that exhibit low sucrose responsiveness. Indeed, highly-responsive flies that did not stop until after the fifth trial failed to show significant savings (F(1, 56) = 2.09, p<.15, η^2^ = .04, Figure 8b), whereas the memory effect was significant for the wildtype flies that made an earlier stop, accounting for 10% of the variability in totalPER (F(1, 82) = 8.88, p<.004, η^2^ = .10, Figure 8a). Notice that because each fly in Figure 8a made a stop before the fifth trial, the control flies’ maintenance of a higher habituation curve beyond the fifth trial indicates that they had a higher probability of resuming responding relative to the memory group that showed equal initial responsiveness.

**Figure 9:**
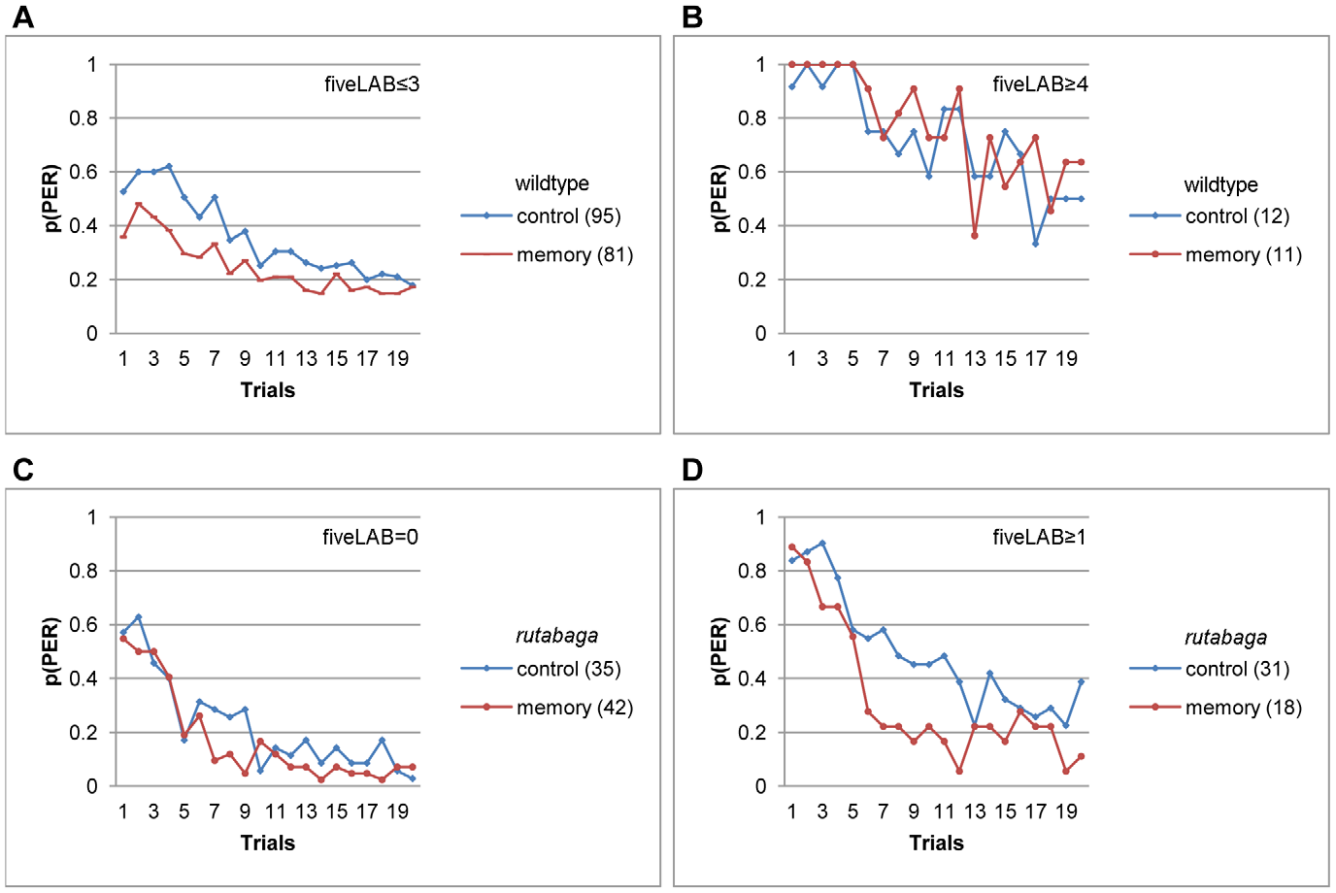
Number of labellar movements during the first five trials is predictive of the expression of 1 hr PER habituation memory. A-B. 1 hour memory of PER habituation for the wildtype flies that emitted 3 or fewer (A), or 4 or more (B) labellar movements during the first five trials. C-D. 1 hour memory for *rutabaga* that produced 0 (C) or 1–5 labellar movements during the first five trials.

Our previous analysis of *de novo* habituation showed that lower numbers of labellar movements during the first five trials were predictive of lower totalPER scores. Similarly, savings due to 1-hr memory were significant for the flies that emitted 3 or less (F(1, 174) = 9.4, p<.003, η^2^ = .05, Figure 9a), but not four or more (F(1, 21) = .55, p<.47, η^2^ = .03, Figure 9b) labellar movements during the first five trials. Taken together, these results suggest that 1 hour memory of PER habituation was either formed, or expressed selectively by the wildtype flies that exhibit lower sucrose responsiveness.

### *rutabaga* Displays Robust Habituation and 1 Hour Memory Following High Initial Sucrose Responsiveness at 2 Hours of Food Deprivation

Interestingly, an analysis of the early responsiveness-state predictors in *rutabaga* revealed a pattern of expression of 1-hr memory complementary to that exhibited by the wildtype flies. For example, *rutabaga* flies that exhibited low sucrose responsiveness, and made their first stop before the fifth trial failed to show a significant memory effect, because both the memory and the control groups had an equally low probability of resuming responding after the first stop (F(1, 81) = 3.94, p<.05, η^2^ = .05, Figure 8c). In contrast, the flies that stopped after the fifth trial revealed the largest 1-hr memory effect we observed in this study (F (1, 26) = 11.09, p<.003, η^2^ = .30, Figure 8d), because the control group resumed responding with a higher probability after a late stop relative to the memory group.

Similarly, only the *rutabaga* flies that emitted one or more labellar movements during the first five trials expressed of 1-hr memory of PER habituation. Figure 9c shows that the memory effect was negligible for *rutabaga* that failed to emit labellar movements during the first five trials (F(1, 75) = 1.91, p<.17, η^2^ = .03). However, the memory effect was significant, and accounted for 9% of the variance in totalPER for *rutabaga* that moved their labella at least once within the first five trials (F(1, 47) = 4.6, p<.04, η^2^ = .09, Figure 9d).

## Discussion

### PER Habituation is State-dependent in Drosophila

Quantitative changes in a continuous variable may sometimes yield state-like changes in behavior [19]–[20]. When that applies, an overall group-average based analysis of data might not be appropriate. Rather, data should be analyzed separately for subjects that display differences in behavioral state.

In this study, when we habituated the wildtype flies *de novo*, the total number of PERs emitted in the session and the habituation curves showed increments in two distinct phases between 1–4 hours of food deprivation. However, these group averages were *not* representative of the response profiles of individual flies. For example, when the habituation curves showed a smooth upshift, individual flies did not exhibit a graded, quantitative increase in responding. Rather, the flies’ response to repeated sucrose stimulation showed discontinuous, state-like transitions under food deprivation [10].

In particular, each fly exhibited one of three distinct patterns of response to repeated sucrose stimulation: Non-responsive, non-habituating, or habituating. Some flies remained non-responsive throughout the session, in spite of the relatively high concentration of sucrose presented (600 mM). In our pilot experiments, we observed that non-responsive flies exhibited poor concentration discrimination because they failed to respond to as high as 1.5 M sucrose. The converse pattern of responsiveness was exhibited by the non-habituating flies. They emitted PERs with non-diminishing vigor throughout the session with the possibility of occasional interruption, and their proboscis extensions were often accompanied with labellar movements [21]. Non-habituating flies displayed remarkably poor concentration discrimination as well, as they failed to respond with lower probability or vigor to lower concentrations of sucrose (data not presented). Finally, a third group of flies initially responded to sucrose, but displayed a reduction in the probability of PER upon repetition of sucrose stimulation. The behavioral pattern of the non-responsive, habituating, and non-habituating flies remained relatively stable, but their relative frequency changed with the period of food deprivation.

### Definition of Responsiveness in Terms of Early Behavioral Parameters

Under the PER habituation paradigm, group indices can be representative of individuals if they are calculated separately for flies in different states of hunger. However, it is not currently feasible to quantify the changes in neuromodulatory transmission that induces state transitions as early as 1–4 hours of food deprivation in *Drosophila* [10]. In the absence of a biological marker, we used early behavioral measures of sucrose responsiveness that could account for a significant amount of variance in the total number of PERs produced by the end of the habituation session. Notice that early response measures do not impose a constraint on subsequent responding by themselves. For example, in principle, flies can make 0–15 PERs in the last 15 trials of the session regardless of the number of PERs they emitted during the first five trials. If the number of PERs in the first five trials is not correlated with a state that mediates the subsequent pattern of responding, the difference in the total number of PERs emitted by the end of the session can be as low as the difference in the number of PERs emitted in the first five. In contrast, if early behavioral measures are reliable indicators of the state of responsiveness, then different levels of responsiveness should predict divergent patterns of habituation during the rest of the session.

When calculated for the first five trials of the session, the number of PERs and labellar movements (fivePER and fiveLAB, respectively), the trial to make the first PER (firstPER), and the trial to make the first stop after emitting at least one PER (firstSTOP) provided reliable indicators of sucrose responsiveness. It is beyond the scope of our results to understand whether the levels of responsiveness that we defined on the basis of statistical criteria would be physiologically distinguishable as well, yet our analyses showed that they can reliably predict variability in PER habituation. When we merged the data across 4 hours of food deprivation, and reanalyzed them to understand the effect of responsiveness, each early response parameter accounted for a much larger amount of variance in total number of PERs than the period of food deprivation. Further, the interaction between the early response parameters and the period of food deprivation was either non-significant, or explained a much smaller amount of variance in totalPER relative to the response index, meaning that the flies that exhibited similar levels responsiveness produced similar number of PERs and followed similar courses of habituation, irrespective of the period of food deprivation.

### Sucrose Responsiveness Predicts the Pattern of Short-term PER Habituation

For the wildtype flies, the period of food deprivation by itself could account for only 18% of the variance in totalPER under *de novo* habituation, because flies showed considerable variability with respect to overall responsiveness at each hour of food deprivation. Early response parameters, on the other hand, accounted for larger amounts of variance in totalPER. For example, when data were collapsed across 4 hours of food deprivation, the number of PERs emitted in the first five trials (fivePER) explained 70% of the variance in totalPER.

Each of the remaining measures of sucrose responsiveness accounted for a lesser amount of variance than fivePER because they were defined for responsive flies only, excluding non-responsive flies from the analysis. For example, the trial to make the first PER (firstPER) accounted for only 23% of the variability in totalPER in wildtype flies. Although a late onset was highly predictive of the absence of a non-habituating state, an early onset by itself had limited power as a predictor because the majority of the flies started responding on the first two trials irrespective of overall sucrose responsiveness. Nevertheless, the trial to make the first stop (firstSTOP), or equivalently, the length of the first uninterrupted sequence of PERs within the first five trials distinguished between the habituating and the non-habituating flies among the early starters, and accounted for 43% of the variance in their totalPERs. This is important because for most flies, the first uninterrupted sequence was also the longest. The high power of the first stop to predict the subsequent number of PERs indicates that if a fly does not cease to respond within the first five trials, it is not likely to habituate later. The converse is also true: If a fly is in a state of responsiveness that is permissive of habituation, it is likely to display an early failure to respond. In other words, when allowed by the internal state of the fly, PER habituation is a rapid process.

Finally, the number of labellar movements that accompanied proboscis extensions within the first five trials also accounted for only 23% of the variance in totalPER in wildtype flies because the majority of the flies emitted 0–1 labellar movements, and formed a heterogenous group with respect to responsiveness. On the other hand, a high incidence of labellar movements was the most reliable indicator of a non-habituating state and the strongest predictor of sustained responding for the wildtype flies. For example, 60% of the flies that emitted 4 or more labellar movements within the first five trials were still responding on the last trial of the session. Extension of the rostrum and movement of the labella are controlled by different motor circuits that can be independently activated [22]. Our results show that the labellar movements have a higher threshold for hunger-driven modulation, and their high incidence is a reliable predictor of stimulus-independent/responsiveness-dependent PER sensitization.

### *rutabaga* are Defective in Attuning the Perceptual Salience of Appetitive Stimuli to Physiological Demands

Even a simple form of behavioral change, like the reduction in reflex probability upon stimulus repetition, involves multiple component processes that can be selectively affected by physiological, stimulus-related, and genetic manipulations. In fact, one of the advantages of the habituation test is that it provides repeated measures of the same response, which enables the dissection of learning independent (e.g., perceptual salience of the stimulus) and learning-dependent (e.g., the trajectory of behavioral change) component processes as they may be differentially affected by the same condition.

For example, the trial to make the first response can be regarded as a measure of the salience of the appetitive stimulus. In fact, under *de novo* habituation, the probability of PER on the first trial is a learning independent measure of the perceptual salience of a novel gustatory stimulus. For the wildtype flies, the probability of PER increased between 1–2 hfd, did not change between 2–3 hfd, and increased again 3–4 hfd, suggesting that the perceptual salience of appetitive stimuli is modulated by at least two different mechanisms with different temporal properties up to 4 hours of food deprivation. For the *rutabaga* flies that are deficient for the Ca^2+^/calmodulin activated adenylate cyclase, the probability of a PER increased between 1–2 hfd, but failed to increase between 3–4 hfd, suggesting that the latter increment involves cAMP signaling. Further, even when their average performance indices (e.g., totalPER) were indistinguishable, individual wildtype and *rutabaga* flies displayed different profiles of responsiveness to repeated sucrose. Most notably, for *rutabaga*, sucrose responsiveness was neither suppressed when sated, nor enhanced when hungry to the same extent that it was for the wildtype flies, suggesting a bidirectional role for cAMP signaling in the attunement of the perceptual salience of appetitive stimuli to physiological state.

Using tip-recording from the labellar L-type sensilla, Motosaka and colleagues [23] reported that the sucrose concentration-response curve of *rutabaga* was indistinguishable from that of the wildtype flies. The similarity of the input functions suggests that the difference between the wildtype flies and *rutabaga* with respect to the perceptual salience of a novel appetitive stimulus is mediated downstream of sensory transduction. A recent study showed that starvation increases dopamine release to the presynaptic terminals of the sugar-sensing neurons, which increases Ca^2+^ influx to the terminals and enhances neurotransmitter release [10]. This process is mediated by the activation of DopEcR which signals via the cAMP pathway [24]. Because the *rutabaga* gene encodes an adenylyl cyclase, the failure of sucrose responsiveness to increase after 3 hours of food deprivation in *rutabaga* flies is likely to have resulted from a deficit in the dopaminergic enhancement of the gain of sugar sensing neurons. Although dopaminergic signaling could not be visualized before 6 hours of food deprivation [10], our behavioral data suggest that its enhancement might be in effect as early as 3 hours of food deprivation.

### Short-term Habituation

*rutabaga,* as well as other mutants of the cAMP pathway have previously been shown to have deficits in the habituation of several reflexes [2]. In contrast, our results showed that although *rutabaga* exhibited a deficit in suppressing responding to novelty or stimulus change, it also displayed a faster rate of decline in PER probability to repeated sucrose presentations. The difference in the pattern of habituation was obvious when *rutabaga* and the wildtype flies were equated for sucrose responsiveness. The wildtype flies displayed an initial increment in PER probability followed by an intermittent pattern of responding, suggestive of a temporary stimulus-driven sensitization process that was eventually overridden by habituation. The stimulus-driven sensitization component was absent for *rutabaga* flies for they displayed an immediate response onset followed shortly by an abrupt cessation of responding.

At a first glance, our observation of a faster rate of habituation for *rutabaga* seems to contradict those from an early study which reported that *rutabaga* was defective in PER habituation to tarsal sucrose [5]. However, a closer inspection reveals a difference of method and terminology, rather than behavioral observations in the two studies. Duerr and Quinn’s [5] habituation protocol involved testing the flies with 100 mM sucrose 10 min after presentation of 4 mM sucrose on the same prothoracic tarsus. *rutabaga* responded to both stimuli with a non-diminished probability of PER, which the authors interpreted as a failure of habituation. However, it is more likely that *rutabaga* responded to the second stimulus with the same probability as it would to a novel stimulus, given the long (10 minute) recovery interval. Indeed, there has been other reports that *rutabaga* showed normal short-term habituation followed by an unusually short latency for spontaneous recovery [25]. Interestingly, Duerr and Quinn [5] also reported that when they used a high concentration sucrose (1 M), *rutabaga* failed to display sensitization when the second stimulus was presented at a shorter (2 minute) inter-stimulus interval. Both our method and results are congruent with what Duerr and Quinn [5] termed the absence of sensitization in *rutabaga*.

A faster rate of decline may also be produced by a faster build up of effector fatigue and/or sensory adaptation. Although both the amount and the temporal precision of transmitter release has been shown to be reduced during repetitive stimulation for *rutabaga* at the neuromuscular junction [26], an earlier decline in PER probability under the current protocol is not likely to have resulted from a faster effector fatigue since *rutabaga* did not show a diminished probability of PER to the stimulation of the contralateral tarsus at the end of the session. However, we cannot eliminate the possibility of receptor adaptation either for the wildtype flies or *rutabaga* in this experiment. Indeed, *rutabaga* has earlier been found to display a faster rate of adaptation to mechanical [27] and olfactory stimuli [28]. On the other hand, *rutabaga* has also been reported to show diminished responsiveness to repeated stimulation in the absence of peripheral adaptation, under two different visually guided orientation paradigms [29]. This centrally mediated effect was correlated with a deficit in local field potentials of the mushroom bodies, which are the sites for multi-modal stimulus integration in the insect brain. In fact, mushroom body lesions have been shown to produce immature habituation to other tarsally-driven stimuli, such as foot shock [30]. Therefore, although we cannot eliminate the possibility of a differential rate of receptor desensitization for *rutabaga*, we suggest that its faster rate of PER habituation might also involve a deficiency in stimulus-driven sensitization and response persistence.

In fact, the faster rate of reduction in sucrose salience in *rutabaga* may contribute to this mutant’s appetitive conditioning defect, regardless of whether it results from an adaptation of sensory receptors, or faster habituation. The neurogenetic analysis of learning and memory in the fruit fly has depended, for the most part, on using the conditioned avoidance paradigm, which measures the difference in the preference of two odors, one of which has previously been paired with an aversive event. Conditioned approach has also been studied by pairing one of the odors with an appetitive, instead of an aversive stimulus [31]. Notice that the training phase in conditioned approach/avoidance paradigms involve repeated presentations of both the conditioned (odor) and the unconditioned stimuli (footshock or sucrose), to which the fly the fly should *not* habituate if associations are to be formed. Multi-modal stimulus associations can be hindered as a result of the reduction in the effectiveness of either the CS or the US by means of habituation or other processes [32]–[33], in addition to a possible defect in coincidence detection [34].

### 1 hour Memory of PER Habituation

Long term PER habituation memory that lasts for several hours would prevent the fly from feeding, so it would not be expected in a small species with low energy reserves. However, when the fly is not hungry, persistence of habituation could be adaptive over short periods because it would disengage the fly from incessant foraging, enabling the initiation of other behaviors in the presence of food. In this experiment, we tested for 1 hr memory of PER habituation at different hours of food deprivation, and found that much like the case with *de novo* habituation, formation and/or expression of 1-hour PER habituation memory depends on initial sucrose responsiveness.

The wildtype flies that displayed low scores for the early response parameters expressed significant savings due to 1 hour memory. For example, when the memory and control flies were equated for making an early stop (before the 5^th^ trial), the memory group completed the session with fewer PERs than the control group. That is, after starting out at the same value, the habituation curves of the memory and control groups diverged after the first stop, because the memory group either had a lower probability of resuming responding, or made fewer PERs upon resumption relative to the *de novo* controls. Similarly, 1 hour memory of PER habituation was evident if the wildtype flies in both the memory and control groups emitted 3 or fewer labellar movements. It should be emphasized that both the memory and the control groups showed marked habituation to a low asymptote when 1 hour memory effect was evident. The converse was also true: The wildtype flies were highly responsive to sucrose (i.e., if they made their first stop after the 5^th^ trial, or emitted 4 or more labellar movements) when the memory effect was not significant. These results suggest that high sucrose responsiveness does not permit the formation or the expression of 1 hour PER habituation memory.

The responsiveness-dependence of PER habituation also explains why 1 hour memory often failed to reach significance, or failed to produce a constant effect when it was tested following different periods of food deprivation. Because the effect of 1 hour memory is very small relative to that of responsiveness, when data are averaged across flies that express different levels of sucrose responsiveness, the variance within groups outweighs the small savings effect under parametric tests. Nevertheless, the relative frequency of flies that express a level of sucrose responsiveness permissive for the expression of 1-hour memory might be high enough to yield significance following shorter periods of food deprivation.

Interestingly, *rutabaga* was not deficient in 1 hour memory of PER habituation, although again, it exhibited a number of differences. Most notably, in contrast to the wildtype flies, *rutabaga* expressed significant savings due to 1 hour memory only if they produced higher numbers of PERs and/or labellar movements in the beginning of the session. That is, when the memory and control groups are equated for initial response patterns, savings was significant only if *rutabaga* made their first stop after the 5^th^ trial, or emitted more than 1 labellar movements during the first five trials. Because *rutabaga* habituates fast, a small memory effect that accelerates habituation was not visible if the flies in the control group were already making few responses in total. Notice that these results do not contradict those obtained from the wildtype flies. In the wildtype flies, both a late stop and a high number of labellar movements were reliable indicators of high responsiveness, and hence predictors of a failure to habituate. Because *rutabaga* showed a defect in the hunger-mediated enhancement of responsiveness, neither a late stop nor a high incidence of labellar movements predicted the occlusion of memory effects by high responsiveness in this mutant.

As an outlook, we suggest the hypothesis that habituation of PER might cause a temporary downregulation of the activity of dopaminergic neurons via a cAMP-independent mechanism. Indeed, the downregulation of dopaminergic transmission causes lower sucrose responsiveness [35]. This effect might then be overridden by the hunger-driven enhancement of dopamine release, which in turn enhances the gain of sugar transmission via cAMP signaling, and sensitizes PER.
